# Reduction of MHC-I expression limits T-lymphocyte-mediated killing of Cancer-initiating cells

**DOI:** 10.1186/s12885-018-4389-3

**Published:** 2018-04-26

**Authors:** Brian J. Morrison, Jason C. Steel, John C. Morris

**Affiliations:** 1 Viral and Rickettsial Diseases Department, Infectious Diseases Directorate, Naval Medical Research Center, Silver Spring, Maryland 20910 USA; 2 Faculty of Medicine, The University of Queensland, Brisbane, Queensland Australia; 3 Gallipoli Medical Research Institute, Greenslopes Private Hospital, Brisbane, Queensland Australia; 4 Division of Hematology-Oncology, University of Cincinnati Cancer Institute, University of Cincinnati, Cincinnati, Ohio 45267 USA

**Keywords:** Cancer stem cells, Cancer-initiating cells, Animal models, Lung cancer, Major histocompatibility complex-I, Cytotoxic T lymphocytes, Interferon-gamma

## Abstract

**Background:**

It has been proposed that cancer establishment, maintenance, and recurrence may be attributed to a unique population of tumor cells termed cancer-initiating cells (CICs) that may include characteristics of putative cancer stem cell-like cells. Studies in lung cancer have shown that such cells can be enriched and propagated in vitro by culturing tumor cells in serum-free suspension as tumorspheres. CICs have been characterized for their phenotype, stem cell-like qualities, and their role in establishing tumor and maintaining tumor growth. Less is known about the interaction of CICs with the immune system.

**Methods:**

We established CIC-enriched tumorspheres from murine TC-1 lung cancer cells, expressing human papillomavirus 16 (HPV-16) E6/E7 antigens, and evaluated their susceptibility to antitumor immune responses both in vitro and in vivo.

**Results:**

TC-1 CICs demonstrated reduced expression of surface major histocompatibility complex (MHC)-I molecules compared to non-CICs. We similarly determined decreased MHC-I expression in five of six human lung cancer cell lines cultured under conditions enriching for CICs. In vivo*,* TC-1 cells enriched for CICs were resistant to human papillomavirus 16 E6/E7 peptide vaccine-mediated killing. We found that vaccinated mice challenged with CIC enriched tumorspheres demonstrated shorter survivals and showed significantly fewer CD8^+^ tumor infiltrating lymphocytes compared to CIC non-enriched challenged mice. Furthermore, cultured cytotoxic T lymphocytes (CTLs) from vaccinated mice demonstrated reduced capacity to lyse TC-1 cells enriched for CICs compared to non-enriched TC-1 cells. Following treatment with IFN-γ, both CIC enriched and non-enriched TC-1 cells expressed similar levels of MHC-I, and the increased MHC-I expression on CICs resulted in greater CTL-mediated tumor lysis and improved tumor-free survival in mice.

**Conclusions:**

These results suggest that the attenuated expression of MHC-I molecules by CICs represents a potential strategy of CICs to escape immune recognition, and that the development of successful immunotherapy strategies targeting CICs may decrease their resistance to T cell-mediated immune detection by enhancing CIC MHC-I expression.

**Electronic supplementary material:**

The online version of this article (10.1186/s12885-018-4389-3) contains supplementary material, which is available to authorized users.

## Background

Solid tumors are made up of a heterogeneous population of cancer cells. It is widely believed that this diversity is a result of a cellular hierarchy, similar to that seen in other tissues, with a compartment of “stem cells” driving the growth of the tumor [1]. Putative “cancer stem cells” or “cancer-initiating cells” (CICs) generate progeny composed of populations of short-term proliferating cells and more differentiated cells that have aberrant differentiation and additional epigenetic changes. CICs share a number of characteristics with normal stem cells, including a capacity for self-renewal, the ability to give rise to differentiated progeny, and increased resistance to DNA damage-induced cell death [2]. CICs are hypothesized to comprise 0.01–10% of cells within the tumor and can be identified based on their stem cell-like characteristics, and the expression of certain cell surface and functional markers.

CICs have been shown to have a number of characteristics that increase their resistance to radiation and chemotherapy, including increased expression of membrane drug transporters that mediate chemotherapy drug efflux, detoxification enzymes that metabolize cytotoxic drugs, up-regulation of anti-apoptotic proteins, increased efficiency of DNA repair, and alterations in cell cycle kinetics [3]. The combination of these factors allows CICs to be intrinsically resistant to traditional treatments potentially leading to enrichment of CICs. Consistent with this, the number of CICs have been shown to be increased in colorectal, glioma and breast cancer mouse xenografts following treatment with chemotherapy and radiation [3–5]. These treatments may initially debulk a tumor of the more “differentiated”, rapidly growing tumor cells, but leaves behind the innately resistant CICs.

Treatments specifically targeting CICs may be a more effective strategy to treat cancer. Therapies that target CICs may eliminate the cells responsible for tumor self-renewal, ultimately leading to tumor regression. As evidence for this, Chen et al., using a model of glioblastoma, showed that chemotherapy treatment of the tumor leads to transient growth arrest, but also leads to the selection of CICs with the capacity to repopulate the tumor. Using a genetic lineage ablation approach to deplete the CICs, they demonstrated increased survival by delaying tumor relapse compared with mice treated with classical chemotherapy alone [6]. Similarly, Nakanishi et al., demonstrated in intestinal cancer that specific targeting of CICs could lead to tumor regression without affecting normal tissue homeostasis [7]. These studies suggest that designing therapies that specifically target CICs can result in antitumor effects and may ultimately have considerable clinical benefit. From this we and others have posited that generating cancer vaccines that specifically target CICs may be a more effective treatment than more general vaccines targeting the bulk of the tumor [2, 8–10]. Very little; however is known about CICs interaction with the immune system and whether they are susceptible to immune-mediated killing.

Herein, we evaluate CICs susceptibility to antitumor immune responses in a murine model. In vitro and in vivo*,* we establish that CICs are intrinsically resistant to cytolytic T-lymphocyte (CTL)-mediated lysis. We identify the down-regulated expression of major histocompatibility class I (MHC-I) molecules on the surface of CICs of both murine and human CICs as a potential factor in the T-cell immune resistance. Furthermore, we demonstrate that MHC-I expression on CICs can be restored through interferon-gamma (IFN-γ) treatment leading to a partial restoration of the sensitivity to CTL killing.

## Methods

### Cell lines

Mouse TC-1 lung cancer cells (American Type Culture Collection (ATCC), Manassas, VA) that express human papillomavirus 16 (HPV-16) E6/E7 were cultured in adherent monolayer conditions, or enriched for CICs in tumorsphere culture as previously described [11–13]. Human lung cancer cell lines A549, Calu-6, H460, H1299, H520, and H522 (ATCC) were cultured as adherent cells in RPMI-1640 (Mediatech, Inc., Manassas, VA) supplemented with 10% fetal calf serum (Invitrogen, Carlsbad, CA) and 1% penicillin G-streptomycin (Invitrogen). Human cells were cultured as CICs under the same conditions as TC-1 cells. Sphere-forming capacity, fold-expansion [14], and the ability for the cells to culture as spheroids for greater than three passages was assessed for each cell line (Table 1). For all of the experiments, passage 2, day 1 spheres represented samples enriched for CICs and matched adherent cultures represented non-CIC controls. Cells were assessed for viability by trypan blue exclusion (Invitrogen). Single cell suspensions were prepared by passage through a 40 μm cell strainer (BD Biosciences, Franklin Lakes, NJ).

**Table 1:** Sphere-forming capacity of selected human lung cancer cell lines

Cell line	Sphere-forming capacity^a^	Long-term proliferation assay^b^	Fold-expansion^c^	Stem cell gene expression^d^		
				OCT4	NANOG	SOX2
A549	+	+	1.2 ± 0.3 (*n* = 15)	45 ± 10	112 ± 33	2 ± 0.4
H460	+	+	3 ± 2.1 (*n* = 24)	38 ± 10	55 ± 7	89 ± 36
H1299	+	+	1.3 ± 0.6 (*n* = 24)	115 ± 35	97 ± 17	79 ± 19
Calu-6	+	–	1.1 ± 0.9 (*n* = 12)	NA	NA	NA
H520	+	–	1 ± 1 (*n* = 20)	NA	NA	NA
H522	+	–	0.6 ± 0.6 (*n* = 17)	NA	NA	NA

^a^Ability to form three-dimensional multicellular spheroids in tumorsphere culture. ^b^Mean fold-expansion > 1 and ability to culture > 3 passages. ^c^Fold-expansion average ± standard deviation. Fold-expansion is calculated over multiple passages. ^d^Gene expression fold-difference assessed by qRT-PCR of CICs (*n* = 2) normalized to GAPDH and standardized to matched non-CIC (*n* = 3) expression ±standard deviation, *P* = 0.005 for all. *NA* = Not applicable

### Real-time reverse transcription-polymerase chain reaction for expression of “stemness” genes

Using matched non-CICs and CICs, total RNA was extracted using PureLink™ RNA mini-kit (Invitrogen). RNA was reverse transcribed in 20 μL using the Verso cDNA kit (Thermo Fisher Scientific, Surrey, UK) and the GeneAmp PCR System 9700 thermocycler (Applied Biosystems, Foster City, CA). Analysis of *OCT4*, *NANOG*, and *SOX2* expression was carried out using Plexor® qPCR System (Promega, Madison, WI) reagents and StemElite™ *Pou5f1/GAPDH*, *NANOG/GAPDH*, or *SOX2/GAPHD* primer pairs (Promega) containing primers for both the gene of interest and the GAPDH gene. Data was collected using the Bio-Rad CFX96™ RT-System (Bio-Rad Laboratories, Hercules, CA) and analyzed using Plexor® analysis software. All real-time RT-PCR results were compiled using three technical repeats for each biological replicate, and two biological repeats for CICs and three biological repeats for non-CICs were conducted for each sample. Data was normalized to endogenous GAPDH for each sample. Samples were standardized to matched non-CICs to compare expression levels.

### Real-time reverse transcription-polymerase chain reaction for HPV-16 E6/E7 gene expression

TC-1 CICs and non-CICs total RNA was extracted using PureLink™ RNA mini-kit (Invitrogen). RNA was reverse transcribed in 20 μL using the Verso cDNA kit (Thermo Fisher Scientific) and the GeneAmp PCR System 9700 thermocycler (Applied Biosystems). Analysis of E6 and E7 expression was carried out using the following primers (Real Time Primers, LLC, Elkins Park, PA): E6-Forward, CTGCAATGTTTCAGGACCCA; E6-Reverse, TCATGTATAGTTGTTTGCAGCTCTGT; E7-Forward, AAGTGTGACTCTACGCTTCGGTT; E7-Reverse, GCCCATTAACAGGTCTTCCAAA. The qPCR was carried out using Bullseye EvaGreen qPCR Mastermix (MidSci, St. Louis, MO). Data was collected using the Bio-Rad CFX96™ RT-System (Bio-Rad Laboratories). All real-time RT-PCR results were compiled using three technical repeats for each biological replicate and three biological repeats were done for each sample. Data was normalized to endogenous murine β-actin (Real Time Primers, LLC) for each sample. Samples were standardized to non-CICs to compare expression levels among samples.

### Flow cytometry for MHC-I

TC-1 cells were stained with fluorescein isothiocyanate (FITC) conjugated anti-mouse H-2Kb (clone AF6–88.5; BD) and mouse (BALB/c) FITC-IgG2a κ (BD) was used as an isotype control. Human cell lines were stained with FITC mouse anti-human HLA-ABC (clone G46–2.6; BD) and a FITC mouse IgG1κ antibody (BD) was used as an isotype control. Data was collected using an Epics XL-MCL flow cytometer (Beckman Coulter, Inc., Brea, CA). Data was analyzed using FlowJo Software (Tree Star, Inc., Ashland, OR). Results represent the change in mean fluorescent intensity (Δ-MFI) ± standard deviation corrected for mean expression of isotype controls and normalized to expression of non-CICs. Positive expression ±standard deviation was similarly corrected for expression of isotype controls. Expression of MHC-I was also assessed following 24 h of exposure to 500 units/mL recombinant mouse IFN-γ (R&D Systems, Minneapolis, MN).

### IFN-γ production by splenocytes on co-culture with CICs and non-CICs

Eight week-old female C57Bl/6 mice (Jackson Laboratory, Bar Harbor, ME) were vaccinated intramuscularly with 50 μg of HPV-16 E7^49–57^ (RAHYNIVTF) peptide (IBA-LifeSciences, Goettingen, Germany) containing 3 μg GM-CSF using the protocol established from [15]. Spleens were harvested 10 days after injection and a single cell suspension of splenocytes was prepared. 1 × 10^6^ splenocytes were co-cultured with 4 × 10^5^ mitomycin C (Sigma, St. Louis, MO) treated TC-1 CICs or non-CICs in 2 mL total of RPMI-1640 media with 10% fetal calf serum in 24-well plates (BD). Mitomycin C treatment, 30 μg/mL, was conducted at 37 °C for 3 h before the cells were washed 4 times with PBS. Cells were then cultured for 3 days before media was collected. IFN-γ concentration was assessed by ELISA (eBioscience, San Diego, CA). Background expression of IFN-γ was controlled for with splenocytes only, media only and TC-1 cells only controls.

### CTL assay

C57Bl/6 mice were vaccinated as described with HPV-16 E7^49–57^ peptide, spleens were harvested on day 10, and a single cell suspension of splenocytes was prepared. CytoTox 96® Non-Radioactive Cytotoxicity Assay (Promega) was used to assess CTL activity. Effector to target (E:T) cell ratios included 100:1, 50:1, 30:1, and 10:1 (*n* = 3). Samples used in these experiments include: (i) CICs; (ii) CICs + IFN-γ treatment; (iii) non-CICs; and (iv) non-CICs + IFN-γ treatment. IFN-γ treatment was for 24-h with the addition of 500 units/mL recombinant mouse IFN-γ. IFN-γ treatment increased MHC-I expression on CICs to levels comparable to non-CICs and non-CICs treated with IFN-γ.

### NK cell assay

Mouse CD49b^+^ NK cells were purified from C57Bl/6 mice splenocytes using a Dynabeads® FlowComp™ Mouse CD49b kit (Invitrogen). Purity of NK cells was assessed using a FITC hamster anti-rat CD49b (clone Ha1/29; BD) antibody and FITC Armenian hamster IgG2*, λ3 (BD) was used as an isotype control. Purity ranged from 92 to 97%. NK cell activity was assessed using CytoTox 96® Non-Radioactive Cytotoxicity Assay (Promega). E:T cell ratios used were 20:1, 10:1, 5:1 and 1:1 (*n* = 4). Samples were as the CTL assay. Results represent mean ± standard deviation of representative experiments. The same set of CICs and non-CICs were used in this assay as were used in the CTL assay.

### Animal experiments

The study protocol was reviewed and approved by the University of Cincinnati Institutional Animal Care and Use Committee (IACUC) in compliance with all applicable Federal regulations governing protection of animals in research. All efforts were made to minimize animal suffering. For all animal studies humane endpoints were utilized to prevent unalleviated pain according to IACUC guidelines and following previously published guidelines [16]. Briefly, tumor burden was measured with calipers and animals were euthanized according to guidelines before tumor size reached the maximum allowed. Regardless of tumor size mice were euthanized if tumors displayed ulceration, if there was distension of covering tissues, or if severe body weight occurred (consistent or rapid weight loss of 15% or greater). Mice were also euthanized if they displayed clinical signs necessitating immediate intervention as per [16]. Euthanasia was performed according to guidelines for carbon dioxide and cervical dislocation euthanasia. No unexpected deaths occurred during this study.

Six to eight week-old female C57Bl/6 mice were obtained from Jackson Laboratories. Mice were vaccinated with the E7^49–57^ peptide as described earlier. Mice (*n* = 12 per group for the vaccinated and 8 per group for the control unvaccinated) received two injections 7 days apart. Tumor cells were implanted subcutaneously from matched CICs or non-CIC cultures at 200,000 cells per mouse seven days after the last vaccination and the mice were followed for tumor-free survival. For the limiting dilution analysis; dilutions of TC-1 cells (120,000, 40,000, 10,000, or 5000) from matched CICs, non-CICs, or CICs or non-CICs treated with 500 units/mL IFN-γ for 24 h were subcutaneously injected into the flanks of C57Bl/6 mice (*n* = 4 for 120,000 cell group and 8 for the three remaining groups). Mice were monitored 3 times per week for time-to-tumor formation. Tumor-free survival up to day 66 was assessed. Kaplan-Meier curves and log-rank survival analysis was conducted to assess differences in tumor-free survival.

### Assessment of tumor-infiltrating lymphocytes

TC-1 non-CIC (*n* = 4, average days after tumor injection, 16.3 ± 5; average tumor size, 31.4 ± 5.8 mm^3^) and CIC (*n* = 5, average days after tumor injection, 19.6 ± 3.8; average tumor size, 31.6 ± 6.7 mm^3^) derived tumors were surgically resected from euthanized mice. Tumor size was determined using the formula 0.5 x length x (width)^2^. Single cell suspensions were prepared by crosshatching tumors with scalpel blades, followed by enzymatic digestion for one hour at 37 °C with collagenase/hyaluronidase (STEMCELL Technologies Inc., Vancouver, Canada). Cells were then passed through a 40 μm cell strainer and washed with PBS. Single cells were then selected for CD45.2 positivity using biotin conjugated anti-mouse CD45.2 (clone 104; eBioscience) and magnetic selection using the CELLection biotin binding kit (Invitrogen). Single cells were then stained with FITC conjugated rat anti-mouse CD49b (clone DX5; Invitrogen) with rat IgM κ (eBioscience) was used as an isotype control, with allophycocyanin (APC) conjugated rat anti-mouse CD8a (clone 53–6.7; BD) with IgG2a, κ (eBioscience) used as an isotype control, and with PE conjugated anti-mouse CD3 (BD) with rat IgG2b, κ (eBioscience) used as an isotype control. Data was collected using BD FACSCalibur flow cytometer (BD). Data was analyzed using FlowJo Software (Tree Star, Inc.).

### Statistical analysis

SigmaPlot 11.0 (Systat Software, Inc., Chicago, IL) was used for statistical analysis. Unpaired two-tailed Student’s *t*-test was used to compare groups, unless otherwise stated. Log-rank survival analysis was conducted to assess differences in tumor-free survival in vivo. Extreme limiting dilution analysis used to assess CIC frequency in vivo as per [17]. The level of significance was set at *P* = 0.05.

## Results

### Cancer-initiating cells are resistant to in vivo vaccine-mediated antitumor effects

We examined whether CICs were resistant to immune-mediated killing following vaccination against an immunodominant antigen present on TC-1 tumor cells. To test this, we used murine TC-1 cancer cells that express HPV-16 E6/E7 [18] cultured in tumorsphere conditions to enrich for CICs or adherent culture conditions as non-enriched (non-CIC) controls [11]. Prior to vaccination we examined E6/E7 mRNA expression by TC-1 CICs and non-CICs and determined that similar levels of the target antigen were expressed (Additional file 1).

**Fig. 1:**
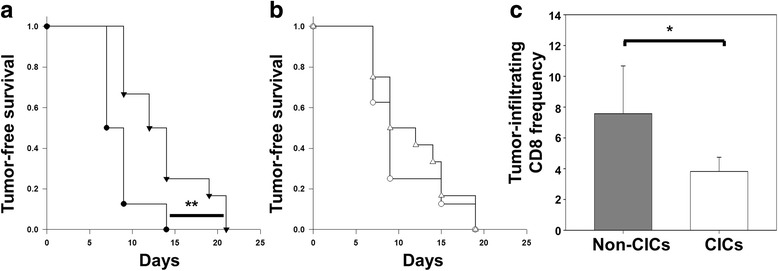
CICs are resistant to in vivo vaccine-mediated antitumor immune responses. Mice (*n* = 8) were subcutaneously injected with 200,000 TC-1 non-CICs or CICs. Other mice were vaccinated (*n* = 12) with HPV-16 E7^49–57^ peptide and subcutaneously injected with non-CICs or CICs. Tumor-free survival was assessed. TC-1 non-CICs (closed circles), CICs (open circles), non-CICs vaccinated (closed triangles), or CICs vaccinated (open triangles). **a** Tumor-free survival was delayed in vaccinated mice receiving non-CICs compared to unvaccinated mice receiving non-CICs. **b** No difference in tumor-free survival was seen for CICs comparing vaccinated to unvaccinated mice. **c** Frequency of tumor-infiltrating CD8^+^ cells is greater in non-CICs (*n* = 4) than in CICs (*n* = 5) derived tumors. Error bars are standard deviation. **P = 0.007, *P = 0.035

Mice vaccinated with HPV-16 E7^49–57^ (RAHYNIVTF) peptide [15] and challenged with TC-1 cancer cells not enriched for CICs had significantly improved tumor-free survival compared to the unvaccinated animals (*P* = 0.007) (Fig. 1a). In contrast, vaccinated mice challenged with preparations enriched for CICs showed no improvement in survival compared to unvaccinated mice (Fig. 1b), indicating that CICs may be intrinsically resistant to vaccine-induced immune responses. Further, when we examined the frequency of tumor infiltrating lymphocytes in these animals we found significantly fewer CD8^+^ cells in the CIC-enriched tumors compared to the non-enriched tumors, *P* = 0.035, (Fig. 1c). In contrast no differences were seen in the overall frequency of tumor-infiltrating NK cells between non-CIC and CIC-derived tumors (data not shown).

### Cancer-initiating cells are resistant to ex vivo CTL responses

**Fig. 2:**
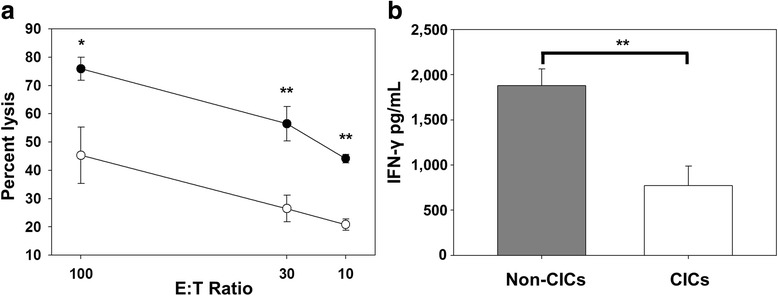
TC-1 CICs are more resistant to CTL-mediated immune responses than non-CICs. **a** CTL assay (representative of three independent experiments) demonstrating non-CICs are more efficiently lysed than CICs by primed splenocytes at each effector to target (E:T) cell ratio (*n* = 3). **b** Enhanced IFN-γ release, as assessed by ELISA, 72-h after co-culture of primed splenocytes and mitomycin C treated non-CICs compared to co-culture with mitomycin C treated CICs, *n* = 8. Error bars are standard deviation. **P ≤ 0.001, *P = 0.004

As further evidence of the innate immune resistance of lung cancer derived CICs, we examined the lytic potential of CICs following co-culture with activated antigen specific cytotoxic lymphocytes in an ex-vivo CTL assay. In these ex-vivo co-culture assays we showed that CTLs derived from mice vaccinated with the HPV-16 E749–57 peptide induced significantly lower levels of lysis of CICs compared to non-CICs at all effector to target cell (E:T) ratios (Fig. 2a). In a separate, but similar, study we found that CTLs co-cultured with CICs induced significantly less IFN-γ secretion than CTLs cultured with non-CICs (Fig. 2b).

### Lung cancer derived cancer-initiating cells express reduced levels of MHC class I

**Fig. 3:**
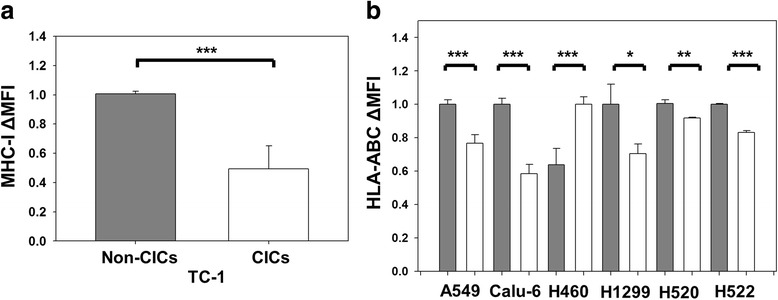
CICs demonstrate reduced expression of MHC-I. **a** MHC-I change in mean fluorescent intensity (Δ-MFI) between non-CICs and CICs for TC-1 cells demonstrates reduced expression of MHC-I on CICs, *n* = 8. **b** HLA-ABC Δ-MFI between non-CICs (filled) and CICs (open) for human lung cancer cell lines demonstrates a decrease in HLA-ABC for CICs compared to non-CICs for five out of six cell lines, *n* = 3–4. Error bars are standard deviation. ***P ≤ 0.001, **P = 0.003, *P = 0.018

Immune recognition of tumor antigens by CTLs is largely mediated through major histocompatibility complex class I (MHC-I) molecules on the surface of tumor cells. MHC-I expression is also a negative regulator of natural killer (NK) cells. CICs isolated from glioblastoma, melanoma, colon and oral cancers have been reported to have reduced expression of MHC-I as a method to escape immune recognition [19–26]. We examined whether TC-1 CICs had reduced surface expression of MHC-I. Compared to non-CICs, TC-1 derived CICs showed decreased expression of MHC-I (*P* ≤ 0.001, *n* = 8) (Fig. 3a). The frequency of CICs (30 ± 17%) expressing MHC-I was also significantly decreased compared to non-CICs (65.3 ± 20.6%; *P* = 0.002, *n* = 8). Next we evaluated whether this was true for human lung cancer derived CICs. We examined MHC-I (HLA-ABC) expression in a panel of human lung cancer cell lines that exhibit sphere-forming capacity, long-term proliferation [14] and a positive fold-expansion consistent with CICs that included A549, Calu-6, H460, H1299, H520, and H522 (Table 1). CICs enriched from five of the six cell lines demonstrated significant reductions in MHC-I expression compared to non-CICs (Fig. 3b). Down-regulation of MHC-I on the surface of both murine and human lung CICs may allow CICs to escape recognition by T-cells.

### Increasing MHC-I on lung cancer derived cancer-initiating cells to enhance T-cell targeting

**Fig. 4:**
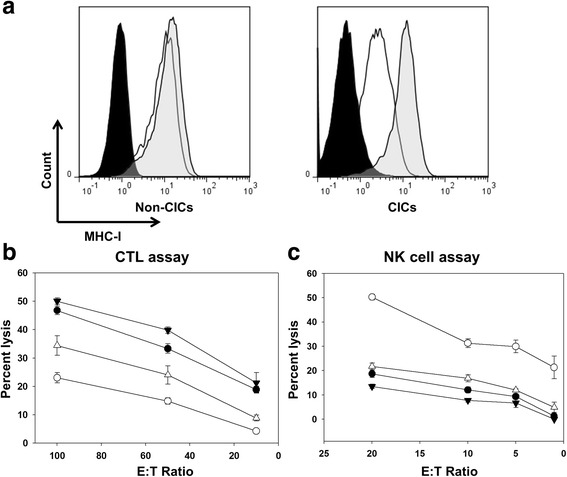
Enhanced MHC-I expression increases targeting of CICs by CTLs, but diminishes NK cell lysis. **a** Flow cytometry histograms (representative of five independent experiments) demonstrating enhanced expression of MHC-I after 24 h treatment with IFN-γ; isotype control (filled), untreated cells (open), cells treated with IFN-γ (tinted). CTL and NK cell assays (representative of two independent experiments) showing percent lysis of target cells co-cultured with either primed splenocytes (**b**) or NK cells (**c**). TC-1 non-CICs (closed circles), CICs (open circles), non-CICs treated with IFN-γ (closed triangles), or CICs treated with IFN-γ (open triangles). CICs are less susceptible to CTL lysis than non-CICs at all effector to target (E:T) cell ratios (*n* = 3) 100; *P* < 0.001, 50; *P* < 0.001, 30; *P* = 0.009, 10; *P* < 0.001. CTL lysis of CICs expressing enhanced MHC-I after IFN-γ treatment did not reach the same level as for non-CICs with and without IFN-γ treatment (E:T ratio 100; *P* < 0.001, 50; *P* = 0.029, 10; *P* < 0.001). CICs are significantly better targeted by NK cells than non-CICs and CICs or non-CICs treated with IFN-γ at all E:T cell ratios (*n* = 4); *P* < 0.001. Error bars are standard deviation

In order for CICs to be targeted by T-cells, developing strategies to increase the MHC-I expression by these cells may be required. IFN-γ has been shown to up-regulate MHC-I expression on cancer cells [27], potentially increasing antigen presentation to T-cells. We assessed expression of MHC-I on TC-1 CICs and non-CICs following treatment with 500 units/mL IFN-γ for 24 h and found that MHC-I was significantly up-regulated on CICs following IFN-γ exposure; *P* ≤ 0.008 (Fig. 4a). MHC-I expression on the non-CIC population was also increased following IFN-γ exposure. The levels of MHC-I expression was similar between CICs and non-CICs following treatment with IFN-γ on day 1 following passage, the time-point cells were used for all assays (Additional file 2).

### Increased MHC-I expression on CICs increases their lysis by CTL

In order to determine whether the increase of MHC-I on the surface of CICs would enhance effector cell recognition and function, we treated CICs with IFN-γ and assessed the activity of CTL derived from HPV-16 E7^49–57^ (RAHYNIVTF) peptide vaccinated mice against TC-1 CICs (Fig. 4b). We demonstrate that increasing expression of MHC-I by IFN-γ leads to significantly greater lysis of CICs compared to CICs without IFN-γ treatment, *P* < 0.001. Lysis was also improved in non-CICs treated with IFN-γ compared to untreated non-CICs. Interestingly, the level of CIC lysis by CTL remained significantly lower than that seen with the non-CICs (*P* ≤ 0.029), even with increased MHC-I expression, indicating that CICs may have other uncharacterized immune suppressive characteristics in addition to their ability to down-regulate MHC-I expression.

Given that increasing MHC-I expression is a negative repressor for NK cell activity, we tested the effect of increasing MHC-I on CICs on NK cell-mediated lysis (Fig. 4c). We showed that TC-1 CICs treated with IFN-γ demonstrated diminished NK cell lysis compared to untreated CICs; *P* < 0.001. Similarly, IFN-γ treated non-CICs with higher MHC-I expression exhibited lower NK cell-mediated lysis than untreated non-CICs. The reduction in NK cell-mediated killing, following IFN-γ treatment and increased MHC-I expression, was not unexpected as NK cell killing is largely dependent on the absence of MHC-I expression on the surface of target cells. However, the loss of NK cell-mediated killing of CICs may be a drawback to increasing MHC-I expression on CICs.

### Increased MHC-I expression on CICs improves tumor-free survival in vivo

**Fig. 5:**
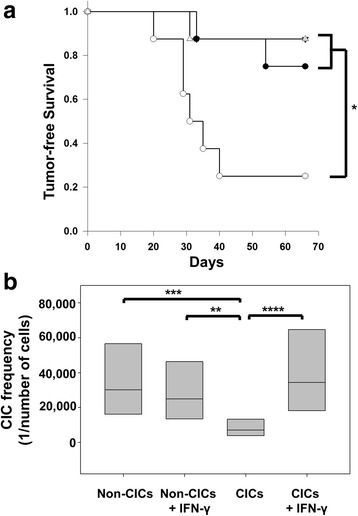
CICs treated with IFN-γ become less tumorigenic. Mice (*n* = 4 for 120,000 group and *n* = 8 for remaining groups) were subcutaneously injected with 120,000, 40,000, 10,000, or 5000 cells from TC-1 non-CICs (closed circles), CICs (open circles), non-CICs treated with IFN-γ (closed triangles), or CICs treated with IFN-γ (open triangles). **a** Tumor-free survival for the 10,000 cell group was assessed and by day 66 was found to be significantly shorter for CICs (*P = 0.027). **b** Confidence interval plot of an extreme limiting dilution analysis to assess CIC frequency per number of cells is shown. Untreated CICs are more tumorigenic than the other three groups (****P < 0.001, ***P = 0.00135, **P = 0.00549)

To assess whether exposure to IFN-γ, subsequent MHC-I up-regulation and potential loss of NK cell-mediated killing would alter the tumorigenicity of CICs; we implanted IFN-γ treated and untreated CICs and non-CICs into immunocompetent mice and followed the mice for tumor formation (Fig. 5a). In line with our previous studies [11], mice injected with untreated CICs had significantly shorter tumor-free survivals than those animals implanted with untreated non-CICs, *P* = 0.027. In contrast, animals transplanted with IFN-γ treated CICs expressing MHC-I had significantly longer survival than the untreated CICs (low MHC-I expression), P = 0.027. There was no difference in tumor-free survival of animals transplanted with non-CICs or IFN-γ treated CICs. The differences in CICs expressing MHC-I with the untreated CICs with low expression of MHC-I suggests that the ability to escape host immune responses is a vital characteristic of CICs. In this model, IFN-γ itself did not affect cell growth or cell viability (Additional file 2).

To confirm that CICs with higher MHC-I expression have reduced cancer-initiating ability we performed an extreme limiting dilution assay [17] in immunocompetent animals. We show that IFN-γ treated CICs, with higher MHC-I levels, require significantly more cells to initiate a tumor in immunocompetent mice than untreated CICs, with lower MHC-I levels (*P* < 0.001) (Fig. 5b). When comparing the high MHC-I expressing IFN-γ treated CICs with non-CICs we found that there was no difference in their tumor-forming capacity. Similarly, there was no difference in the tumor initiating capacity of IFN-γ treated non-CICs with that of the untreated non-CICs. Our results provide evidence for the importance of MHC-I in the escape of CICs from host immune responses allowing for the initiation tumors.

## Discussion

Putative cancer stem cell-like cells, cells having phenotypic and functional properties similar to stem cells, have been shown to form multicellular three-dimensional spheres in vitro when grown in non-adherent and serum-free culture conditions [11–13]. This sphere assay allows for investigation of phenotypic properties associated with cancer stem cell-like cells, and a mathematical interpretation allowing for assessment of symmetric division expansion rate allows for a functional assessment of cancer stem cell-like cells [14]. Further assessment of tumor-initiating capacity in vivo allows for determination of CICs. In many cases CICs and cancer stem cell-like cells are terms that have been used to describe the same subset of cells, but specifically CICs should demonstrate capacity to establish tumors while cancer stem cell-like cells demonstrate properties of stem cells. Unique features of cancer stem cell-like cells and/or CICs can be potentially exploited as specific targets for lung tumor control [2, 10].

With the advent of new checkpoint inhibitor treatments showing efficacy in lung cancer, understanding the interaction of the immune system and cancer has become increasingly important [28]. In this study, we examined the interplay of the immune system with CICs, an important population of tumors cells responsible for tumor initiation, maintenance, progression, and therapeutic resistance. We have previously demonstrated that TC-1 murine lung cancer cells grown under tumorsphere culture conditions are enriched in cancer stem cell-like functional and phenotypic qualities and that CIC frequency is enhanced [11]. Using this murine transplantable tumor cell line in immunocompetent mice, herein we showed that tumors derived from inoculums enriched for CICs were resistant to an acquired tumor vaccine immune response. Vaccinated mice implanted with CICs showed no inhibition of tumor growth and the CICs themselves were resistant to T-cell-mediated lysis. The majority of CIC studies rely upon immunocompromised xenograft models of cancer initiation, which cannot adequately examine the interplay between the host immune system and tumor. We previously reported the importance of examining tumor initiation in immune competent hosts [11] as this better models the situation in patients with cancer, where tumor cells must interact with host immune cells to prevent tumor recognition and elimination.

The MHC-I complex on the surface of cells plays an important role in antigen recognition by cytotoxic T-cells. In this study, we demonstrated that putative lung CICs of both human and murine origin exhibit reduced levels of MHC-I compared to non-CICs. The cancer-initiating capacity of the human CICs, grown as spheres, compared to the non-CICs, grown as adherent cells, investigated in this study remain to be further elucidated. However, the human CICs demonstrate characteristics in line with putative cancer stem cell-like cells, and similar to the TC-1 cells demonstrated reduction in MHC-I expression for five out of six cell lines. Our results were in line with other studies that have demonstrated that CICs isolated from other tumor types including glioblastoma, melanoma, colon and oral cancers of human origin also exhibit reduced expression of MHC-I [19–26]. The reduction of MHC-I by the CICs may therefore be a common strategy used by CICs to escape immune recognition.

In order to show the importance of MHC-I in immune evasion we treated CICs with IFN-γ to increase MHC-I expression. In our lung cancer cell line, IFN-γ increased MHC-I expression without altering CIC growth or survival. The increase in MHC-I expression on the lung cancer cells was; however, able to partially sensitize the CICs to T-cell-mediated recognition and lysis. Di Tomaso et al. demonstrated that tumor stem cells from glioblastoma patients cultured as spheres in the presence of IFN-γ also displayed increased MHC-I expression [21]. Similarly, in head and neck cancer, Liao et al. showed that MHC-I can be increased with IFN-γ in CICs leading to improvements in T-cell recognition and lysis [29].

Interestingly, despite a lack of complete sensitization of CICs to T-cell killing, CICs treated with IFN-γ to increase MHC-I expression, prior to animal implantation, resulted in a significant improvement in tumor-free survival in mice. This result was mirrored in the limited dilution study in immune competent mice where we showed that significantly more IFN-γ treated cells are required for tumor initiation, indicating the importance of MHC-I down-regulation in the tumor initiating process in vivo. Future studies will be needed to investigate the expression of MHC-I, Ki-67, Caspase-3, NK cell markers, and activated T-cell markers in tumor tissue comparing CICs to non-CICs and the effect of IFN-γ on cancer markers and immune cell infiltration of tumors.

In this study, we also showed that the addition of IFN-γ resulted in an inverse killing relationship by NK cells. Here, increasing MHC-I expression on the surface of the lung CICs resulted in decreased killing of CICs by NK cells. While this is in line with the known inhibitory effects of MHC-I on NK cells, it is contradictory to the results published by Wu et al., who reported that the treatment of glioma CICs with IFN-γ, while increasing MHC-I, also increased NK cell-mediated lysis [26]. Future studies will be needed to specifically assess the role of T cells and NK cells for controlling CIC in vivo, in particular in regards to how MHC-I expression may influence the balance of CTL vs. NK cell killing. However, the increased susceptibility of lung derived CICs to NK cell lysis, seen in our study, may provide a rationale for NK cell based therapies to target residual MHC-I low expressing CICs following treatment to debulk the tumor. Interleukin-15 or IL-2-activated NK cells may be a more efficient means of targeting clinically relevant CICs, ultimately leading to improved treatment for patients.

## Conclusions

Our study demonstrates the importance of decreased MHC-I expression by lung CICs as a method to subvert the immune system and enhance tumorigenicity. Down-regulation of MHC-I expression results in reduced CTL killing, but may lead to CICs being targeted by NK cells. Assessing ways to enhance either MHC-I expression by tumor cells or to enhance NK cell killing of MHC-I deficient/low expressing cells may allow for better immunological targeting of lung CICs.

## Additional files

Additional file 1: Expression of HPV-16 E6/E7 for TC-1 CICs and non-CICs. Real-time qPCR quantitation of matched non-CICs and CICs for E6 (**A**) and E7 (**B**) expression. Relative fold-increase compared to non-CICs and normalized for expression of β-actin. No significant difference in expression was found. Error bars are standard deviation. NS = Not significant. (TIF 112 kb)

Additional file 2: Fold-expansion and viability in vitro is not affected by treatment with IFN-γ. (**A**) Relative change in fold-expansion, and (**B**) viability of TC-1 non-CICs and CICs treated with IFN-γ was calculated compared to expression of untreated matched cells at each time point. Cells were exposed to 500 units/mL IFN-γ for 24 h. Cells were then washed and day 1 fold-expansion was calculated. CICs were re-plated in fresh media and assessed at day 4 and day 6 for MHC-I expression by (**C**) frequency positive and (**D**) change in mean fluorescent intensity (Δ-MFI). Non-CICs were cultured in a similar manner, but passaged again at day 4 when they reached confluence. No differences were seen for relative change in fold-expansion or viability following treatment with IFN-γ compared to no treatment. MHC-I positivity and Δ-MFI decreased over time for CICs treated with IFN-γ. At each time point CICs treated with IFN-γ expressed more MHC-I than the untreated CICs. Non-CICs treated with IFN-γ expressed more MHC-I than untreated non-CICs at day 1 and day 4, but were not significantly different at day 6. Δ-MFI and positivity for MHC-I decreased over time for non-CICs treated with IFN-γ. ***P < 0.001, **P = 0.004, *P = 0.011. Error bars are standard deviation. NS = Not significant. (TIF 421 kb)

## Abbreviations

CICs: Cancer-initiating cells
CTLs: Cytotoxic T lymphocytes
E:T: Effector to target cell
FITC: Fluorescein isothiocyanate
HPV: Human papillomavirus
IFN-γ: Interferon-gamma
MHC-I: Major histocompatibility class I
NK: Natural killer
