# Supplementary figures and images for: Reduction of MHC-I expression limits T-lymphocyte-mediated killing of Cancer-initiating cells
Source: BMC Cancer. 2018 Apr 26;18:469. doi: 10.1186/s12885-018-4389-3 (PMC5918869; doi:10.1186/s12885-018-4389-3)

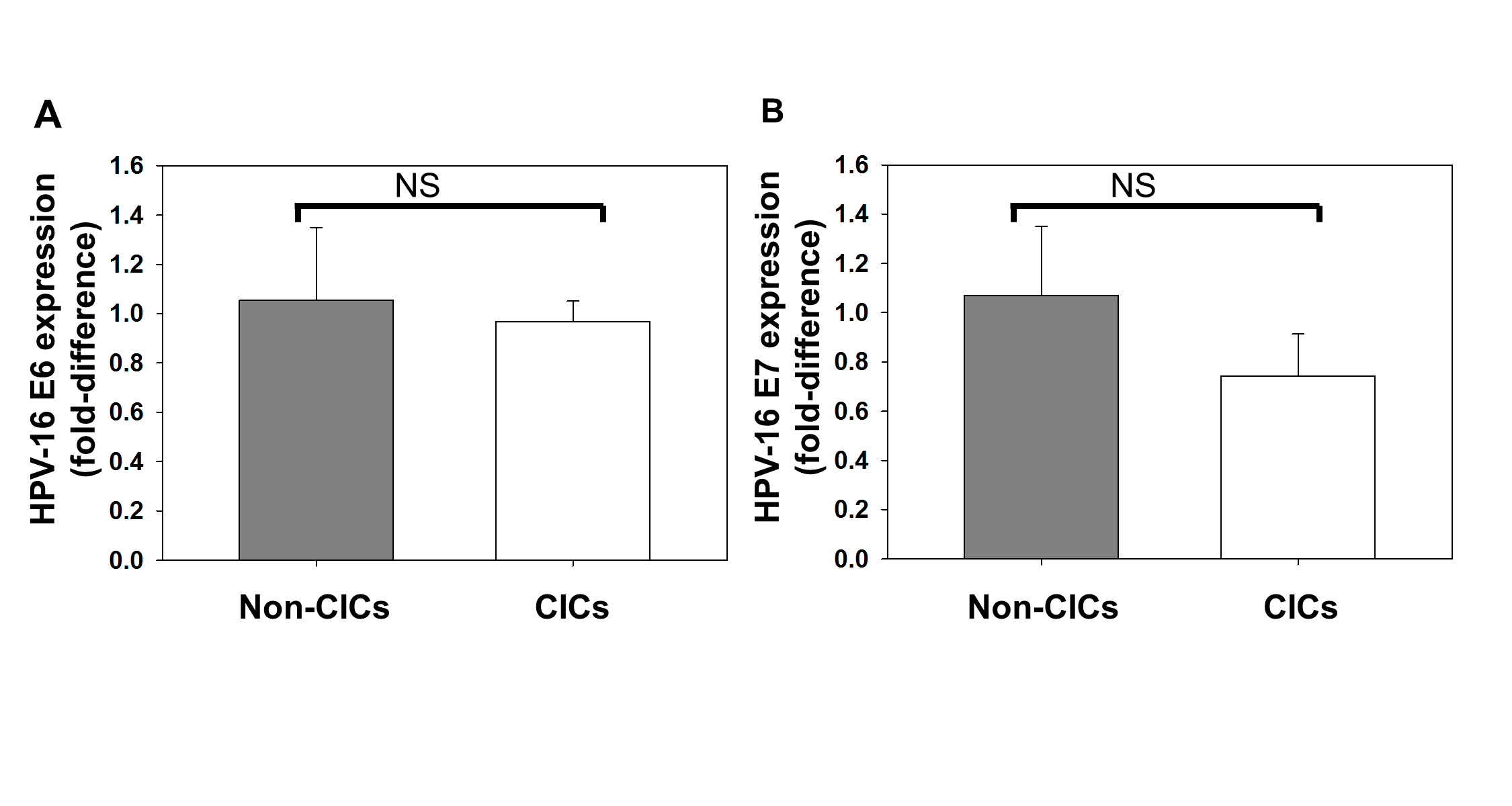

Supplement: Supplementary file 1 — Expression of HPV-16 E6/E7 for TC-1 CICs and non-CICs. Real-time qPCR quantitation of matched non-CICs and CICs for E6 (A) and E7 (B) expression. Relative fold-increase compared to non-CICs and normalized for expression of β-actin. No significant difference in expression was found. Error bars are standard deviation. NS = Not significant. (TIF 112 kb) [file 12885_2018_4389_MOESM1_ESM.tif]

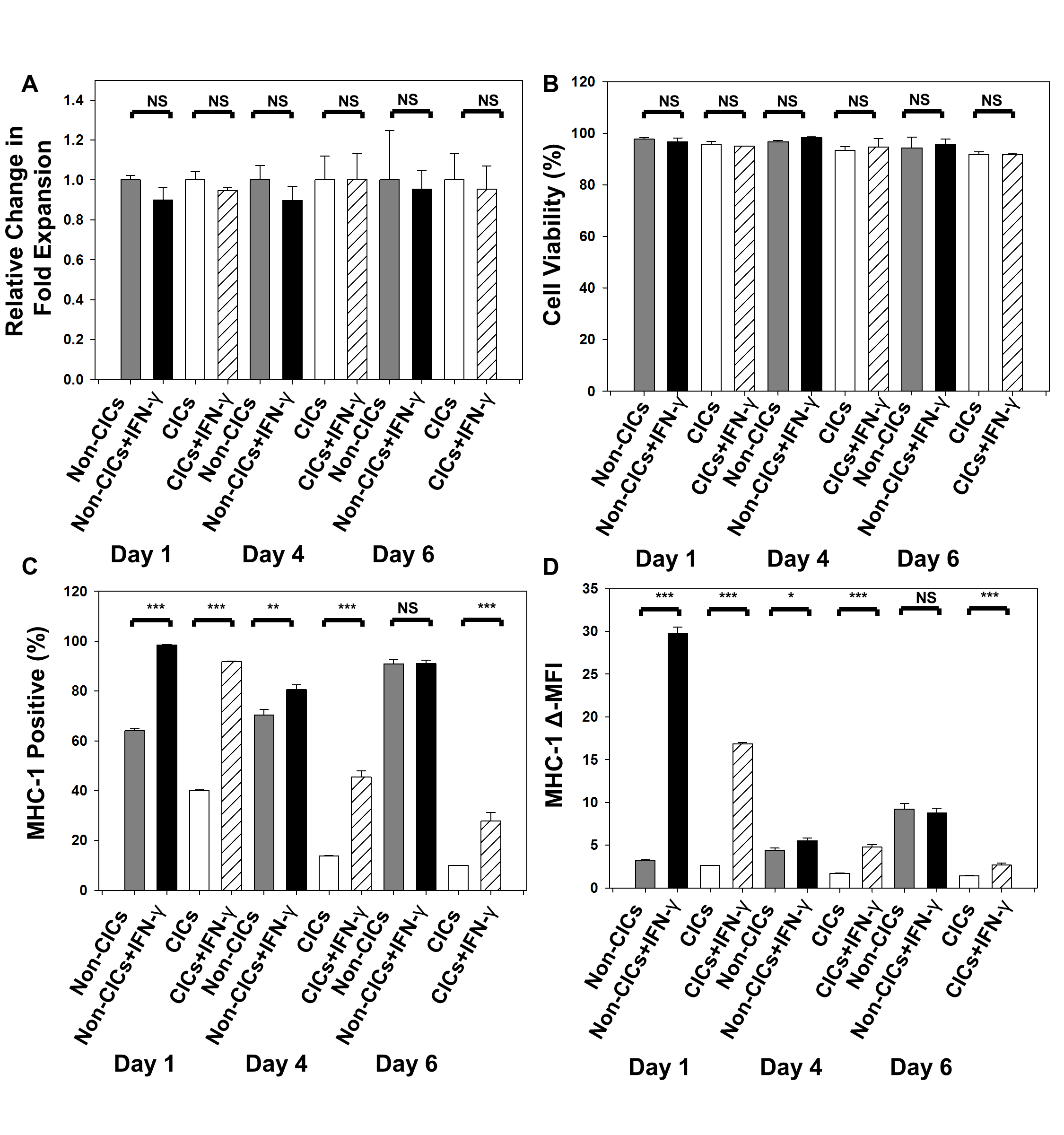

Supplement: Supplementary file 2 — Fold-expansion and viability in vitro is not affected by treatment with IFN-γ. (A) Relative change in fold-expansion, and (B) viability of TC-1 non-CICs and CICs treated with IFN-γ was calculated compared to expression of untreated matched cells at each time point. Cells were exposed to 500 units/mL IFN-γ for 24 h. Cells were then washed and day 1 fold-expansion was calculated. CICs were re-plated in fresh media and assessed at day 4 and day 6 for MHC-I expression by (C) frequency positive and (D) change in mean fluorescent intensity (Δ-MFI). Non-CICs were cultured in a similar manner, but passaged again at day 4 when they reached confluence. No differences were seen for relative change in fold-expansion or viability following treatment with IFN-γ compared to no treatment. MHC-I positivity and Δ-MFI decreased over time for CICs treated with IFN-γ. At each time point CICs treated with IFN-γ expressed more MHC-I than the untreated CICs. Non-CICs treated with IFN-γ expressed more MHC-I than untreated non-CICs at day 1 and day 4, but were not significantly different at day 6. Δ-MFI and positivity for MHC-I decreased over time for non-CICs treated with IFN-γ. ***P < 0.001, **P = 0.004, *P = 0.011. Error bars are standard deviation. NS = Not significant. (TIF 421 kb) [file 12885_2018_4389_MOESM2_ESM.tif]
